# Supplementary material for: Acting without considering personal costs signals trustworthiness in helpers but not punishers
Source: Commun Psychol. 2024 May 24;2:47. doi: 10.1038/s44271-024-00092-7 (PMC11332106; doi:10.1038/s44271-024-00092-7)
Supplement: Supplementary file 1 — Supplementary Information [file 44271_2024_92_MOESM1_ESM.pdf]

SUPPLEMENTARY METHODS

Qualtrics Survey Content Examples

1.1 Punishment Cost Checking (1.2a)

Start of Block: Information and consent

This study has been approved by the UCL Ethics Board. Project ID: ICN-PWB-NR-23-10-20A.

The study is being run by Nicole Engeler (nicole.engeler.19@ucl.ac.uk) under the supervision of Professor Nichola Raihani (n.raihani@ucl.ac.uk) in the Department of Experimental Psychology at UCL.

In this study, you will play one or two games. Your decisions during these games will affect your bonus payment and the bonus payment of other Prolific players.

The study is estimated to last around X minutes.

All data will be handled according to the General Data Protection Regulations and will be kept anonymous. Only researchers working with the Raihani Lab will have access to datafiles containing identifying information (e.g. Prolific ID). Completely de-identified datafiles (e.g. no Prolific ID) will be shared online in accordance with open-science practices. If you do not consent to de-identified data being shared, please do not take part in this study. If you do not wish to complete this study, please return your submission.

Local Data Protection Privacy Notice

The controller for this project will be University College London (UCL). The UCL Data Protection Officer provides oversight of UCL activities involving the processing of personal data, and can be contacted at data-protection@ucl.ac.uk

The lawful basis that will be used to process your personal data is: 'Public task'

Your personal data will be processed so long as it is required for the research project. We will anonymise or pseudonymise the personal data you provide, and will endeavour to minimise the processing of personal data wherever possible.

If you are concerned about how your personal data is being processed, or if you would like to contact us about your rights, please contact UCL in the first instance at data-protection@ucl.ac.uk.

If you do not wish to participate, or if you change your mind during the course of the study, please return your submission.

Ethics Officer: ethics@ucl.ac.uk

Please only consent to taking part in this study if you agree with ALL of the following statements:
(1) I have read the information above and understand I can email the researchers with any questions.
(2) I understand that my personal information will be used for the purposes explained to me. I understand that according to data protection legislation, 'public task' will be the lawful basis for processing.
(3) I understand that all personal information will remain confidential and that my data gathered in this study will be stored anonymously and securely. It will not be possible to identify me in any publications.
(4) I understand that my anonymised research data may be shared with, and used by, others for future research (no one will be able to identify you when these data are shared).
(5) I understand that I am free to withdraw from the study without penalty if I so wish, simply by returning my submission.

- ◯ I **consent** to take part in this study. (1)
- ◯ I do **NOT consent** to take part in this study. (0)

Skip To: End of Survey If Please only consent to taking part in this study if you agree with ALL of the following statement... = I do NOT consent to take part in this study.

Please enter your Prolific ID

____

Start of Block: Player A – Game 1

In addition to the base payment (£0.90) for participating in this study, you can earn a **bonus** based on your interaction with other Prolific participants during *two different games*.

Although some aspects of the two games are similar, in the first game you will be one of THREE players, and in the second game you will be one of TWO players.

GAME 1

GAME 1 is a three-player game. **You will be Player 3.**

Player 1 and **Player 2** have already read all the rules and made their decisions, and are now waiting for yours.

In GAME 1, each player starts with the following amounts:
Player 1: £0.10
Player 2: £0.00

You: £0.10

Player 1 has to choose how much, if anything, to send to **Player 2**.
Any amount sent to **Player 2** is tripled (e.g. for every 1p **Player 1** sends, **Player 2** receives 3p).
Player 2 then has to choose how much, if anything, to return to **Player 1**.

For instance, if **Player 1** sends £0.10 to Player 2, **Player 2** receives £0.30.
If **Player 2** returns half, both players earn £0.15 and both have more than **Player 1** started with.
If **Player 2** returns nothing, Player 2 keeps £0.30 and **Player 1** earns no bonus.

Players can also send or return any intermediate amount.

Player 1 chose to send all £0.10 to Player 2, and therefore Player 2 received £0.30.

Player 2 chose to keep everything (£0.30) and to return nothing (£0.00).

As a result, Player 1 will now receive no bonus (£0.00) and Player 2 will receive a bonus of £0.30.

You (**Player 3**) start this game with £0.10, and **can pay a cost to punish Player 2 by removing some of their bonus**. If you decide to punish, **Player 2's bonus will be reduced by £0.15**. (**Player 1**'s bonus will not be affected by your decision.)

However, **you DO NOT know how much the cost to yourself is**, only that it is somewhere between £0.00 and £0.10.

If you choose to punish **Player 2**, you will keep whatever remains of your £0.10 bonus.
If you choose NOT to punish, you will keep all £0.10.

Example: If the cost to punish **Player 2** turned out to be £0.05, **you** would keep £0.05.
Example: If the cost to punish **Player 2** turned out to be £0.10, **you** would keep £0.00.
Example: If the cost to punish **Player 2** turned out to be £0.00, **you** would keep £0.10.

While you currently do NOT know how much it would cost you to punish, you can request to find out before deciding whether to punish.

So, **you** must decide:
1) Whether or not to find out how much it would cost you to punish **Player 2**
2) Whether or not to punish **Player 2**

Please answer the following questions, to make sure you understand GAME 1. **You MUST answer ALL questions correctly to receive your bonus!**

Imagine that you are deciding whether or not to punish **Player 2**. Which *decision* that **you** make will likely result in the **highest** possible bonus for **you**?

- You deciding to punish (0)
- You deciding NOT to punish (1)
- Either decision could result in a higher bonus than the other decision, it solely depends on what the cost of punishing is (0)

Imagine that you are deciding whether or not to punish **Player 2**. Which *decision* that **you** make will result in the **lowest** possible bonus for **Player 2**?

- You deciding to punish (1)
- You deciding NOT to punish (0)
- Which decision results in the lowest bonus depends on what the cost of punishing is (0)

Imagine that you are deciding whether or not to punish **Player 2**. Which *decision* that **you** make will result in the **highest** possible bonus for **Player 1**?

- You deciding to punish (0)
- You deciding NOT to punish (0)
- Your decision does not affect Player 1's bonus (1)

What are the two choices that you have?

◯ Whether to find out the cost of punishing, and whether to punish Player 2 (1)

◯ Whether to punish Player 2 if the cost of punishing is large, and whether to punish Player 2 if the cost of punishing is small (0)

◯ Whether to punish Player 2, and how much to punish Player 2 (0)

Start of Block: Player A – Game 2

GAME 2

After GAME 1, you will play GAME 2, which is a **two-player** game.

You will play GAME 2 with a NEW player who did NOT participate in GAME 1.

You will be the "**Receiver**". The new player will be the "Sender". There will be no further players.

In GAME 2:
- The Sender starts with £0.10
- The Sender chooses how much, if anything, to send to you (the **Receiver**)
- Any money sent to **you** is tripled: for every 1p the Sender sends, **you** receive 3p
- **You** then choose how much, if anything, to return to the Sender. You can return nothing, any intermediate amount, or everything.

If the Sender sends all £0.10, **you** will receive £0.30. If **you** return half of the £0.30, **you** and the Sender will both earn £0.15, and will both have more than the Sender started with.

But if **you** return £0.00, you will earn £0.30, while the Sender will earn £0.00.

*So, the Sender can gain or lose money by sending you money, depending on how much **you** return.*

Please answer the following questions, to make sure you understand GAME 2. **You MUST answer ALL questions correctly to receive your bonus!**

Imagine that the Sender is deciding how much to send to you. Which of the ***Sender's** decisions* will result in the **Sender** earning the highest payoff?

- The Sender deciding to send nothing (0)
- The Sender deciding to send everything (0)
- Which decision results in the highest payoff depends on how much you decide to return to the Sender (1)

Imagine that you are deciding how much to return to the Sender. Which of ***your** decisions* will result in **you** earning the highest payoff?

- You deciding to return nothing (1)
- You deciding to return everything (0)
- Which decision results in the highest payoff depends on how much the Sender sent you (0)

Did the **Sender** participate in GAME 1?

- Yes (0)
- No (1)

Start of Block: Player A Game 2 OBSERVABLE

Although the **Sender** did NOT participate in GAME 1, the **Sender** can base the decision of how much money to send to you on how **you** behaved in GAME 1.

Specifically, the Sender can base this decision on whether you decided to punish Player 2, as well as whether you decided to find out the cost of punishing.

Please answer the following question, to make sure you understand. You MUST answer ALL questions correctly to receive your bonus!

When deciding how much to send to **you**, what information can the **Sender** base this decision on?

- Nothing (0)
- Whether you punished Player 2 (0)
- Whether you decided to find out the cost of punishing (0)
- Whether you punished Player 2, AND whether you decided to find out the cost of punishing (1)

GAME 1

Punishing entails **you** paying a cost to remove £0.15 from **Player 2**'s £0.30 bonus. The cost is somewhere between £0.00 and £0.10, but you do not know exactly how much.

NOTE: This study involves REAL PEOPLE. *You really do have the chance to find out the cost of punishing, and the Sender really does get to base their sending decision on whether you decide to find out*.

Would you like to find out the cost of punishing Player 2?

- Yes (1)
- No (0)

Start of Block: Checked (Player A)

The cost of punishing is £0.05.

Right now **you** have a £0.10 bonus. You know that punishing would cost **you** £0.05 to reduce **Player 2**'s £0.30 bonus by £0.15.

Would you like to pay the cost to punish Player 2?

- ◯ Yes (1)
- ◯ No (0)

Start of Block: Not Checked (Player A)

You decided not to find out what the cost of punishing is.

X→

Right now **you** have a £0.10 bonus. Punishing means paying a cost to reduce **Player 2**'s £0.30 bonus by £0.15. The cost is somewhere between £0.00 and £0.10.

Would you like to pay the cost to punish Player 2?

- ◯ Yes (1)
- ◯ No (0)

Start of Block: Player A Game 2 HIDDEN

Although the Sender did NOT participate in GAME 1, the Sender can base the decision of how much money to send to you on how **you** behaved in GAME 1.

Specifically, the Sender can base this decision on whether you decided to punish Player 2 (but *NOT* on whether you decided to find out the cost of punishing).

When deciding how much to send to you, what information can the Sender base this decision on?

- Nothing (0)
- Whether you punished Player 2 (1)
- Whether you decided to find out the cost of punishing (0)
- Whether you punished Player 2, AND whether you decided to find out the cost of punishing (0)

GAME 1

Punishing entails **you** paying a cost to remove £0.15 from **Player 2**'s £0.30 bonus. The cost is somewhere between £0.00 and £0.10, but you do not know exactly how much.

NOTE: This study involves REAL PEOPLE. *You really do have the chance to find out the cost of punishing.*

Would you like to find out the cost of punishing Player 2?

- Yes (1)
- No (0)

Start of Block: Player A Game Decisions

GAME 2

The Sender will decide how much of their £0.10 to send to you.
You will decide how much to return to the Sender.

Specifically, you will decide the percentage of pence you would like to return.

For example, if **you** decide to return 40% of the pence you receive...

- If the Sender sends £0.00 (and you receive £0.00) **you** will return £0.00
- If the Sender sends £0.10 (and you receive £0.30) **you** will return £0.12

REMINDER: This study involves REAL PEOPLE, and your decision will determine how much bonus you and the Sender actually receive.

What percentage would you like to return?

	0 10 20 30 40 50 60 70 80 90 100
Percentage to return ()	

Start of Block: Player B – Game 1

In addition to the base payment (£0.90) for participating in this study, you can earn a **bonus** based on your interaction with other Prolific participants during *two different games*.

You will ONLY take part in the second game, and will NOT play in the first game. However, we would like you to read about and understand both games.

GAME 1

GAME 1 is a three-player game.

Each player starts with the following amounts:
Player 1: £0.10
Player 2: £0.00
Player 3: £0.10

Player 1 has to choose how much, if anything, to send to **Player 2**.
Any amount sent to **Player 2** is tripled (e.g. for every 1p **Player 1** sends, **Player 2** receives 3p).
Player 2 then has to choose how much, if anything, to return to **Player 1**.

For instance, if **Player 1** sends £0.10 to Player 2, **Player 2** receives £0.30. If **Player 2** returns half, both players earn £0.15 and both have more than **Player 1** started with.
If **Player 2** returns nothing, then **Player 2** keeps £0.30 and **Player 1** earns no bonus.
Players can also send or return any intermediate amount.

***Player 1** and **Player 2** have already read all the rules and made their decisions.*

GAME 1

Player 1 chose to send all £0.10 to Player 2, and therefore Player 2 received £0.30.

Player 2 chose to keep everything (£0.30) and to return nothing (£0.00).

As a result, Player 1 will now receive no bonus (£0.00) and Player 2 will receive a bonus of £0.30.

Player 3 now has the chance to pay a cost to remove some of Player 2's bonus. If **Player 3** punishes, *Player 2's bonus will be reduced by £0.15*. (**Player 1**'s bonus will not be affected by Player 3's decision.)

Player 3 DOES NOT know how much this cost is, only that it is somewhere between £0.00 and £0.10.

If **Player 3** chooses to punish **Player 2** by paying the cost, **Player 3** only gets to keep whatever remains of their initial £0.10 bonus.
If **Player 3** chooses NOT to punish, Player 3 gets to keep all £0.10.

Example: If the cost to punish **Player 2** turned out to be £0.05, **Player 3** would keep £0.05.
Example: If the cost to punish **Player 2** turned out to be £0.10, **Player 3** would keep £0.00.
Example: If the cost to punish **Player 2** turned out to be £0.00, **Player 3** would keep £0.10.

While Player 3 initially does NOT know how much it costs them to punish, Player 3 can request to find out before deciding whether to punish.

So, **Player 3** must decide:
1) Whether or not to find out how much it would cost them to punish **Player 2**
2) Whether or not to punish **Player 2**

Please answer the following questions, to make sure you understand GAME 1. **You MUST answer ALL questions correctly to receive your bonus!**

Imagine that Player 3 is deciding whether or not to punish **Player 2**. Which of ***Player 3**'s decisions* will likely result in the **highest** possible bonus for **Player 3**?

- Player 3 deciding to punish (0)
- Player 3 deciding NOT to punish (1)
- Either decision could result in a higher bonus than the other decision, it solely depends on what the cost of punishing is (0)

Imagine that Player 3 is deciding whether or not to punish **Player 2**. Which of **Player 3**'s decision will result in the **lowest** possible bonus for **Player 2**?

- Player 3 deciding to punish (1)
- Player 3 deciding NOT to punish (0)
- Which decision results in the lowest bonus depends on what the cost of punishing is (0)

Imagine that Player 3 is deciding whether or not to punish **Player 2**. Which of **Player 3**'s decision will result in the **highest** possible bonus for **Player 1**?

- Player 3 deciding to punish (0)
- Player 3 deciding NOT to punish (0)
- Player 3's decision does not affect Player 1's bonus (1)

What are the two choices that **Player 3** has?

- Whether to find out the cost of punishing, and whether to punish Player 2 (1)
- Whether to punish Player 2 if the cost of punishing is large, and whether to punish Player 2 if the cost of punishing is small (0)
- Whether to punish Player 2, and how much to punish Player 2 (0)

Start of Block: Player B – Game 2

GAME 2

GAME 2 is a two-player game. *You will play this game with **Player 3** from GAME 1.*

You will be the "**Sender**". **Player 3** will be the "**Receiver**".

In GAME 2:
- **You** (the Sender) start with £0.10
- **You** then choose how much, if anything, to send to the **Receiver**
- Any money **you** send to the **Receiver** is tripled: for every 1p **you** send, the **Receiver** receives 3p
- The **Receiver** then chooses how much, if anything, to return to you. The Receiver can return nothing, any intermediate amount, or everything.

If **you** send all £0.10, the **Receiver** will receive £0.30. If the **Receiver** returns half of the £0.30, **you** and the **Receiver** will both earn £0.15, and will both have more than **you** started with.

But if the **Receiver** returns nothing, they will earn £0.30, while **you** will earn nothing.

*So, **you** can gain or lose money by sending money to the Receiver, depending on how much the **Receiver** returns.*

Please answer the following questions, to make sure you understand GAME 2. **You MUST answer ALL questions correctly to receive your bonus!**

Imagine that you are deciding how much to send to the **Receiver**. Which of ***your** decisions* will result in the **highest** possible bonus for **you**?

○ You deciding to send nothing (0)

○ You deciding to send everything (0)

○ Which decision results in the highest bonus depends on how much the Receiver decides to return to you (1)

Imagine that the Receiver is deciding how much to return to you. Which of the Receiver's decisions will result in the **highest** possible bonus for the Receiver?

- The Receiver deciding to return nothing (1)
- The Receiver deciding to return everything (0)
- Which decision results in the highest bonus depends on how much you sent to the Receiver (0)

Did the Receiver participate in GAME 1?

- Yes (1)
- No (0)

Start of Block: Player B Game 2 OBSERVABLE

GAME 2

You currently have a £0.10 bonus.

You will now decide how much, if anything, to send to the Receiver.

You can base this decision on how the Receiver behaved as Player 3 in GAME 1. Specifically, you can base your decision both on whether the Receiver decided to find out what the cost of punishing was, and whether the Receiver decided to punish Player 2.

In GAME 1, the cost of punishing turned out to be £0.05. So, punishing entailed Player 3 sacrificing £0.05 to remove £0.15 from Player 2. But Player 3 only knew what the cost of punishing was if they decided to find out.

In GAME 2, you will make ***four different choices***, one for each of the possible Player 3 decisions. Your choice that matches with the decision the Receiver actually made in GAME 1 will count for your bonus.

NOTE: This study involves REAL PEOPLE, and your decisions will determine how much bonus you and the Receiver actually receive.

Start of Block: Bdec1OBS

How many pence would you like to send to a **Receiver** who, as **Player 3** in GAME 1...

Chose TO punish **Player 2**, WITHOUT finding out how much it would cost them to punish?

	0	2	4	6	8	10
Pence to send ()						

Start of Block: Bdec2OBS

How many pence would you like to send to a **Receiver** who, as **Player 3** in GAME 1...

Chose NOT to punish **Player 2**, WITHOUT finding out how much it would cost them to punish?

	0	2	4	6	8	10
Pence to send ()						

Start of Block: Bdec3OBS

How many pence would you like to send to a **Receiver** who, as **Player 3** in GAME 1...

Chose TO punish **Player 2**, WITH finding out how much it would cost them to punish?

	0	2	4	6	8	10
Pence to send ()						

Start of Block: Bdec4OBS

How many pence would you like to send to a **Receiver** who, as **Player 3** in GAME 1...

Chose NOT to punish **Player 2**, WITH finding out how much it would cost them to punish?

	0	2	4	6	8	10
Pence to send ()						

Start of Block: Player B Game 2 HIDDEN

GAME 2

You currently have a £0.10 bonus.

You will now decide how much, if anything, to send to the Receiver.

You can base this decision on how the Receiver behaved as Player 3 in GAME 1. Specifically, you can base your decision on whether the Receiver decided to punish Player 2.

In GAME 1, the cost of punishing turned out to be £0.05. So, punishing entailed Player 3 sacrificing £0.05 to remove £0.15 from Player 2. But Player 3 only knew what the cost of punishing was if they decided to find out.

In GAME 2, you will make **two different choices**, one for each of the possible Player 3 decisions. Your choice that matches with the decision the Receiver actually made in GAME 1 will count for your bonus.

NOTE: This study involves REAL PEOPLE, and your decisions will determine how much bonus you and the Receiver actually receive.

Start of Block: Bdec1hid

How many pence would you like to send to a Receiver who, as Player 3 in GAME 1...

Chose TO punish Player 2?

	0	2	4	6	8	10
Pence to send ()						

Start of Block: Bdec2hid

How many pence would you like to send to a Receiver who, as Player 3 in GAME 1...

Chose NOT to punish Player 2?

	0	2	4	6	8	10
Pence to send ()						

Start of Block: demographics

Thank you!

Lastly, please provide your demographic information.

What is your gender?

- ◯ Male (1)
- ◯ Female (2)
- ◯ Other (3)
- ◯ Prefer not to say (4)

---

*

What is your age in years?

____

Start of Block: debrief

Debrief

Player 3 / the Receiver and the Sender really are Prolific participants, and your decisions really do determine your bonus and the bonus of the other player. However, Player 1 and Player 2 in the first game are not real.

Please email nicole.engeler.19@ucl.ac.uk or message on Prolific if you would like further information.

Thank you for your participation!

Please advance to the next page to submit your responses.

1.2 Punishment Personal Cost Decision Time Players A (1.2b)

Start of Block: Information and consent

This study has been approved by the UCL Ethics Board. Project ID: ICN-PWB-NR-23-10-20A.

The study is being run by Nicole Engeler (nicole.engeler.19@ucl.ac.uk) under the supervision of Professor Nichola Raihani (n.raihani@ucl.ac.uk) in the Department of Experimental Psychology at UCL.

In this study, you will play one or two games. Your decisions during these games will affect your bonus payment and the bonus payment of other Prolific players.

The study is estimated to last around X minutes.

All data will be handled according to the General Data Protection Regulations and will be kept anonymous. Only researchers working with the Raihani Lab will have access to datafiles containing identifying information (e.g. Prolific ID). Completely de-identified datafiles (e.g. no Prolific ID) will be shared online in accordance with open-science practices. If you do not consent to de-identified data being shared, please do not take part in this study. If you do not wish to complete this study, please return your submission.

Local Data Protection Privacy Notice

The controller for this project will be University College London (UCL). The UCL Data Protection Officer provides oversight of UCL activities involving the processing of personal data, and can be contacted at data-protection@ucl.ac.uk

The lawful basis that will be used to process your personal data is: 'Public task'

Your personal data will be processed so long as it is required for the research project. We will anonymise or pseudonymise the personal data you provide, and will endeavour to minimise the processing of personal data wherever possible.

If you are concerned about how your personal data is being processed, or if you would like to contact us about your rights, please contact UCL in the first instance at data-protection@ucl.ac.uk.

If you do not wish to participate, or if you change your mind during the course of the study, please return your submission.

Ethics Officer: ethics@ucl.ac.uk

X→

Please only consent to taking part in this study if you agree with ALL of the following statements:
(1) I have read the information above and understand I can email the researchers with any questions.
(2) I understand that my personal information will be used for the purposes explained to me. I

understand that according to data protection legislation, 'public task' will be the lawful basis for processing.
(3) I understand that all personal information will remain confidential and that my data gathered in this study will be stored anonymously and securely. It will not be possible to identify me in any publications.
(4) I understand that my anonymised research data may be shared with, and used by, others for future research (no one will be able to identify you when these data are shared).
(5) I understand that I am free to withdraw from the study without penalty if I so wish, simply by returning my submission.

○ I **consent** to take part in this study. (1)

○ I do **NOT consent** to take part in this study. (0)

Skip To: End of Survey If Please only consent to taking part in this study if you agree with ALL of the following statement... = I do NOT consent to take part in this study.

Please enter your Prolific ID

____

Start of Block: Game 1 comprehension

In addition to the base payment (£0.90) for participating in this study, you can earn a **bonus** based on your interaction with other Prolific participants during *two different games*.

Although some aspects of the two games are similar, in the first game you will be one of THREE players, and in the second game you will be one of TWO players.

GAME 1

GAME 1 is a three-player game. **You will be Player 3.**

Player 1 and Player 2 have already read all the rules and made their decisions, and are now waiting for yours.

In GAME 1, each player starts with the following amounts:
Player 1: £0.10
Player 2: £0.00
You: £0.10

Player 1 has to choose how much, if anything, to send to Player 2.
Any amount sent to Player 2 is tripled (e.g. for every 1p Player 1 sends, Player 2 receives 3p).
Player 2 then has to choose how much, if anything, to return to Player 1.

For instance, if Player 1 sends £0.10 to Player 2, Player 2 receives £0.30.
If Player 2 returns half, both players earn £0.15 and both have more than Player 1 started with.
If Player 2 returns nothing, Player 2 keeps £0.30 and Player 1 earns no bonus.

Players can also send or return any intermediate amount.

Timer1 Timing

First Click (1)

Last Click (2)

Page Submit (3)

Click Count (4)

GAME 1

Player 1 chose to send all £0.10 to Player 2, and therefore Player 2 received £0.30.

Player 2 chose to keep everything (£0.30) and to return nothing (£0.00).

As a result, Player 1 will now receive no bonus (£0.00) and Player 2 will receive a bonus of £0.30.

You (Player 3) start this game with £0.10, and can pay a cost to punish **Player 2** by removing some of their bonus. If you decide to punish, ***Player 2's bonus will be reduced by £0.15***. (Player 1's bonus will not be affected by your decision.)

However, ***right now you do not know how much the cost to yourself is***, only that it is somewhere between £0.00 and £0.10

If **you** choose to punish **Player 2**, you will keep whatever remains of your £0.10 bonus.
If you choose NOT to punish, you will keep all £0.10.

Example: If the cost to punish **Player 2** turned out to be £0.05, **you** would keep £0.05.
Example: If the cost to punish **Player 2** turned out to be £0.10, **you** would keep £0.00.
Example: If the cost to punish **Player 2** turned out to be £0.00, **you** would keep £0.10.

*While you currently do not know how much it costs **you** to punish **Player 2**, we will tell you before you have to decide whether to punish.*

Please answer the following questions, to make sure you understand GAME 1. **You MUST answer ALL questions correctly to receive your bonus!**

Imagine that you are deciding whether or not to punish **Player 2**. Which of ***your*** *decisions* will likely result in the **highest** possible bonus for **you**?

- You deciding to punish (0)
- You deciding NOT to punish (1)
- Either decision could result in a higher bonus than the other decision, it solely depends on what the cost of punishing is (0)

Imagine that you are deciding whether or not to punish **Player 2**. Which of ***your*** *decisions* will result in the **lowest** possible bonus for **Player 2**?

- You deciding to punish (1)
- You deciding NOT to punish (0)
- Which decision results in the lowest bonus depends on what the cost of punishing is (0)

Imagine that you are deciding whether or not to punish **Player 2**. Which of ***your*** *decisions* will result in the **highest** possible bonus for **Player 1**?

- You deciding to punish (0)
- You deciding NOT to punish (0)
- Your decision does not affect Player 1's bonus (1)

If you decide to punish **Player 2**, what is the cost to you?

- 0 pence (0)
- 5 pence (0)
- 10 pence (0)
- You do not know yet, but you will find out later (1)

Start of Block: Game 2 comprehension

Timer2 Timing

First Click (1)

Last Click (2)

Page Submit (3)

Click Count (4)

GAME 2

After GAME 1, you will play GAME 2, which is a two-player game.

You will play GAME 2 with a NEW player who did NOT participate in GAME 1.

You will be the “**Receiver**". The new player will be the "**Sender**". There will be no further players.

In GAME 2:
- The **Sender** starts with £0.10
- The **Sender** chooses how much, if anything, to send to you (the **Receiver**)
- Any money sent to **you** is tripled: for every 1p the **Sender** sends, **you** receive 3p
- **You** then choose how much, if anything, to return to the **Sender**. You can return nothing, any intermediate amount, or everything.

If the **Sender** sends all £0.10, **you** will receive £0.30. If **you** return half of the £0.30, **you** and the **Sender** will both earn £0.15, and will both have more than the **Sender** started with.

But if **you** return £0.00, you will earn £0.30, while the **Sender** will earn £0.00.

*So, the **Sender** can gain or lose money by sending you money, depending on how much you return.*

Please answer the following questions, to make sure you understand GAME 2. **You MUST answer ALL questions correctly to receive your bonus!**

X→

Imagine that the Sender is deciding how much to send to you. Which of the ***Sender's*** *decisions* will result in the **Sender** earning the highest payoff?

- ◯ The Sender deciding to send nothing (0)
- ◯ The Sender deciding to send everything (0)
- ◯ Which decision results in the highest payoff depends on how much you decide to return to the Sender (1)

X→

Imagine that you are deciding how much to return to the Sender. Which of ***your*** *decisions* will result in **you** earning the highest payoff?

- ◯ You deciding to return nothing (1)
- ◯ You deciding to return everything (0)
- ◯ Which decision results in the highest payoff depends on how much the Sender sent you (0)

X→

Did the **Sender** participate in GAME 1?

- ◯ Yes (0)
- ◯ No (1)

Start of Block: Observable

Timer3obs Timing

First Click (1)

Last Click (2)

Page Submit (3)

Click Count (4)

Soon you will be told the cost of punishing. We will then record **how long it takes you to decide whether or not to punish Player 2**.

Although the Sender did NOT participate in GAME 1, they can base the decision of how much money to send to you in GAME 2 on how you behaved in GAME 1.

Specifically, the Sender can base this decision on whether you decided to punish Player 2, and on how long it took you to decide whether to punish Player 2.

Please answer the following question, to make sure you understand. *You MUST answer ALL questions correctly to receive your bonus!*

When deciding how much to send to you, what information can the Sender base this decision on?

- Nothing (0)
- Whether you punished Player 2 (0)
- How long it took you to decide whether to punish Player 2 (0)
- Whether you punished Player 2, AND how long it took you to decide whether to punish Player 2 (1)

GAME 1

*On the next screen, we will tell you how much it costs to punish. **You** then decide whether to punish **Player 2**.*

NOTE: This study involves REAL PEOPLE. In GAME 2, the Sender really does get to base their sending decision on whether you decide to punish in GAME 1, and on how long it takes you to decide whether to punish.

We will record how long you spend on the next screen.

Start of Block: Pun Decision

pun timer Timing

First Click (1)

Last Click (2)

Page Submit (3)

Click Count (4)

X→

Punishing costs £0.05.

Punishing means sacrificing £0.05 of your £0.10 bonus to remove £0.15 from Player 2's £0.30 bonus.

Would you like to punish Player 2?

- Yes (1)
- No (0)

Start of Block: Hidden

Timer3hid Timing

First Click (1)

Last Click (2)

Page Submit (3)

Click Count (4)

Soon you will be told the cost of punishing. We will then record **how long it takes you to decide whether or not to punish Player 2.**

Although the Sender did NOT participate in GAME 1, they can base the decision of how much money to send to you in GAME 2 on how you behaved in GAME 1.

Specifically, the Sender can base this decision on whether you decided to punish Player 2 (but NOT on how long it took you to decide whether to punish Player 2).

Please answer the following question, to make sure you understand. *You MUST answer ALL questions correctly to receive your bonus!*

X→

When deciding how much to send to you, what information can the Sender base this decision on?

- ○ Nothing (0)
- ○ Whether you punished Player 2 (1)
- ○ How long it took you to decide whether to punish Player 2 (0)
- ○ Whether you punished Player 2, AND how long it took you to decide whether to punish Player 2 (0)

GAME 1

*On the next screen, we will tell you how much it costs to punish. **You** then decide whether to punish **Player 2**.*

NOTE: This study involves REAL PEOPLE. In GAME 2, the Sender really does get to base their sending decision on whether you decide to punish in GAME 1.

We will record how long you spend on the next screen.

Start of Block: Return Decision

GAME 2

The Sender will decide how much of their £0.10 to send to you.
You will decide how much to return to the Sender.

Specifically, you will decide the percentage of pence you would like to return.

For example, if you decide to return 40% of the pence you receive...
• If the Sender sends £0.00 (and you receive £0.00) you will return £0.00
• If the Sender sends £0.10 (and you receive £0.30) you will return £0.12

REMINDER: This study involves REAL PEOPLE, and your decision will determine how much bonus you and the Sender actually receive.

What percentage would you like to return?

	0 10 20 30 40 50 60 70 80 90 100
Percentage to return ()	

Start of Block: demographics

Thank you!

Lastly, please provide your demographic information.

What is your gender?

○ Male (1)

○ Female (2)

○ Other (3)

○ Prefer not to say (4)

*

What is your age in years?

____

Start of Block: debrief

Debrief

The Sender really is another Prolific participant, and your decisions really do determine your bonus and the bonus of the other player. However, Player 1 and Player 2 in the first game are not real.

Please email nicole.engeler.19@ucl.ac.uk or message on Prolific if you would like further information.

Thank you for your participation!

Please advance to the next page to submit your responses.

1.3 Punishment Personal Cost Decision Time Players B (1.2b)

Start of Block: Information and consent

This study has been approved by the UCL Ethics Board. Project ID: ICN-PWB-NR-23-10-20A.

The study is being run by Nicole Engeler (nicole.engeler.19@ucl.ac.uk) under the supervision of Professor Nichola Raihani (n.raihani@ucl.ac.uk) in the Department of Experimental Psychology at UCL.

In this study, you will play one or two games. Your decisions during these games will affect your bonus payment and the bonus payment of other Prolific players.

The study is estimated to last around X minutes.

All data will be handled according to the General Data Protection Regulations and will be kept anonymous. Only researchers working with the Raihani Lab will have access to datafiles containing identifying information (e.g. Prolific ID). Completely de-identified datafiles (e.g. no Prolific ID) will be shared online in accordance with open-science practices. If you do not consent to de-identified data being shared, please do not take part in this study. If you do not wish to complete this study, please return your submission.

Local Data Protection Privacy Notice

The controller for this project will be University College London (UCL). The UCL Data Protection Officer provides oversight of UCL activities involving the processing of personal data, and can be contacted at data-protection@ucl.ac.uk

The lawful basis that will be used to process your personal data is: 'Public task'

Your personal data will be processed so long as it is required for the research project. We will anonymise or pseudonymise the personal data you provide, and will endeavour to minimise the processing of personal data wherever possible.

If you are concerned about how your personal data is being processed, or if you would like to contact us about your rights, please contact UCL in the first instance at data-protection@ucl.ac.uk.

If you do not wish to participate, or if you change your mind during the course of the study, please return your submission.

Ethics Officer: ethics@ucl.ac.uk

X→

Please only consent to taking part in this study if you agree with ALL of the following statements:
(1) I have read the information above and understand I can email the researchers with any questions.
(2) I understand that my personal information will be used for the purposes explained to me. I understand that according to data protection legislation, 'public task' will be the lawful basis for processing.
(3) I understand that all personal information will remain confidential and that my data gathered in this study will be stored anonymously and securely. It will not be possible to identify me in any publications.
(4) I understand that my anonymised research data may be shared with, and used by, others for future research (no one will be able to identify you when these data are shared).
(5) I understand that I am free to withdraw from the study without penalty if I so wish, simply by returning my submission.

○ I **consent** to take part in this study. (1)

○ I do **NOT consent** to take part in this study. (0)

Skip To: End of Survey If Please only consent to taking part in this study if you agree with ALL of the following statement... = I do NOT consent to take part in this study.

Please enter your Prolific ID

____

Start of Block: Game 1 Instructions

In addition to the base payment (£0.90) for participating in this study, you can earn a **bonus** based on your interaction with other Prolific participants during *two different games*.

You will ONLY take part in the second game, and will NOT play in the first game. However, we would like you to read about and understand both games.

GAME 1

GAME 1 is a three-player game.

Each player starts with the following amounts:
Player 1: £0.10
Player 2: £0.00
Player 3: £0.10

Player 1 has to choose how much, if anything, to send to **Player 2**.
Any amount sent to **Player 2** is tripled (e.g. for every 1p **Player 1** sends, **Player 2** receives 3p).

Player 2 then has to choose how much, if anything, to return to **Player 1**.

For instance, if **Player 1** sends £0.10 to Player 2, **Player 2** receives £0.30. If **Player 2** returns half, both players earn £0.15 and both have more than **Player 1** started with.
If **Player 2** returns nothing, then **Player 2** keeps £0.30 and **Player 1** earns no bonus. Players can also send or return any intermediate amount.

Player 1 *and* ***Player 2*** *have already read all the rules and made their decisions.*

GAME 1

Player 1 chose to send all £0.10 to Player 2, and therefore Player 2 received £0.30.

Player 2 chose to keep everything (£0.30) and to return nothing (£0.00).

As a result, Player 1 will now receive no bonus (£0.00) and Player 2 will receive a bonus of £0.30.

Player 3 now has the chance to punish **Player 2** by removing some of their bonus. If **Player 3** punishes, **Player 2**'s bonus will be reduced by £0.15. (**Player 1**'s bonus will not be affected by Player 3's decision.)

At first, **Player 3** does not know how much this cost is, only that it is somewhere between £0.00 and £0.10.

However, right before we ask Player 3 to decide whether to punish Player 2, we tell Player 3 that the cost is £0.05. Therefore, punishing entails **Player 3** sacrificing £0.05 of their £0.10 bonus to remove £0.15 from **Player 2**'s £0.30 bonus.

After we reveal this information to Player 3, we ***record how long Player 3 takes to decide whether or not to punish Player 2.***

Please answer the following questions, to make sure you understand GAME 1. **You MUST answer ALL questions correctly to receive your bonus!**

Imagine that Player 3 is deciding whether or not to punish **Player 2**. Which of ***Player 3****'s decisions* will result in the **highest** possible bonus for **Player 3**?

- Player 3 deciding to punish (0)
- Player 3 deciding NOT to punish (1)
- Either decision could result in a higher bonus than the other decision, it solely depends on what the cost of punishing is (0)

Imagine that Player 3 is deciding whether or not to punish **Player 2**. Which of ***Player 3****'s decisions* will result in the **lowest** possible bonus for **Player 2**?

- Player 3 deciding to punish (1)
- Player 3 deciding NOT to punish (0)
- Which decision results in the lowest bonus depends on what the cost of punishing is (0)

Imagine that Player 3 is deciding whether or not to punish **Player 2**. Which of ***Player 3****'s decisions* will result in the **highest** possible bonus for **Player 1**?

- Player 3 deciding to punish (0)
- Player 3 deciding NOT to punish (0)
- Player 3's decision does not affect Player 1's bonus (1)

What does **Player 3** know about the cost of punishing **Player 2**?

- Player 3 never knows the exact cost of punishing (but does know that the cost is between 0p and 10p) (0)
- Player 3 always knows that the exact cost of punishing is 5p (0)
- Player 3 initially does not know the exact cost of punishing, but finds out that it is 5p right before deciding whether to punish Player 2 (1)

Start of Block: Game 2 Instructions

GAME 2

GAME 2 is a two-player game. *You will play this game with **Player 3** from GAME 1*.

You will be the "**Sender**". **Player 3** will be the "**Receiver**".

In GAME 2:
• **You** (the Sender) start with £0.10
• **You** then choose how much, if anything, to send to the Receiver
• Any money **you** send to the **Receiver** is tripled: for every 1p **you** send, the **Receiver** receives 3p
• The **Receiver** then chooses how much, if anything, to return to **you**. The Receiver can return nothing, any intermediate amount, or everything.

If **you** send all £0.10, the **Receiver** will receive £0.30. If the **Receiver** returns half of the £0.30, **you** and the **Receiver** will both earn £0.15, and will both have more than you started with.

But if the **Receiver** returns nothing, they will earn £0.30, while **you** will earn nothing.

*So, **you** can gain or lose money by sending money to the Receiver, depending on how much the **Receiver** returns.*

Please answer the following questions, to make sure you understand GAME 2. **You MUST answer ALL questions correctly to receive your bonus!**

X→
Imagine that you are deciding how much to send to the **Receiver**. Which of ***your*** *decisions* will result in the **highest** possible bonus for **you**?

○ You deciding to send nothing (0)

○ You deciding to send everything (0)

○ Which decision results in the highest bonus depends on how much the Receiver decides to return to you (1)

Imagine that the Receiver is deciding how much to return to you. Which of the ***Receiver's** decisions* will result in the **highest** possible bonus for the **Receiver**?

- The Receiver deciding to return nothing (1)
- The Receiver deciding to return everything (0)
- Which decision results in the highest bonus depends on how much you sent to the Receiver (0)

Did the **Receiver** participate in GAME 1?

- Yes (1)
- No (0)

Start of Block: Observable

GAME 2

Reminder: **Player 3** was told the cost of punishing is £0.05 right before deciding whether to punish **Player 2**, and then we recorded how long **Player 3** took to decide.

In GAME 1, half of **Player 3**s took X.XX seconds or longer to decide whether to punish. We will refer to these Player 3s as "taking a long time to decide". The other half of **Player 3**s took less than X.XX seconds to decide. We will refer to these Player 3s as "taking a short time to decide".

You currently have a £0.10 bonus. You will now decide how much, if anything, to send to the **Receiver**.

You can base this decision on how the **Receiver** behaved as **Player 3** in GAME 1.

Specifically, you can base your decision on whether the Receiver decided to punish Player 2, and how long the Receiver took to decide.

In GAME 2, **you** will make ***four different choices***, one for each of the possible **Player 3** actions. **Your** choice that matches the action the **Receiver** actually took in GAME 1 will count for your bonus.

NOTE: This study involves REAL PEOPLE, and your decisions will determine how much bonus you and the Receiver actually receive.

Start of Block: dec1OBS

How many pence would you like to send to a **Receiver** who, as **Player 3** in GAME 1...

Chose TO punish **Player 2**, after taking a SHORT time to decide?

	0	2	4	6	8	10
Pence to send ()						

Start of Block: dec2OBS

How many pence would you like to send to a **Receiver** who, as **Player 3** in GAME 1...

Chose NOT to punish **Player 2**, after taking a SHORT time to decide?

	0	2	4	6	8	10
Pence to send ()						

Start of Block: dec3OBS

How many pence would you like to send to a **Receiver** who, as **Player 3** in GAME 1...

Chose TO punish **Player 2**, after taking a LONG time to decide?

	0	2	4	6	8	10
Pence to send ()						

Start of Block: dec4OBS

How many pence would you like to send to a **Receiver** who, as **Player 3** in GAME 1...

Chose NOT to punish **Player 2**, after taking a LONG time to decide?

	0	2	4	6	8	10
Pence to send ()						

Start of Block: Hidden

GAME 2

Reminder: **Player 3** was told the cost of punishing is £0.05 right before deciding whether to punish **Player 2**, and then we recorded how long **Player 3** took to decide.

In GAME 1, half of **Player 3s** took X.XX seconds or longer to decide whether to punish. We will refer to these Player 3s as "taking a long time to decide". The other half of **Player 3s** took less than X.XX seconds to decide. We will refer to these Player 3s as "taking a short time to decide".

You currently have a £0.10 bonus. You will now decide how much, if anything, to send to the **Receiver**.

You can base this decision on how the **Receiver** behaved as **Player 3** in GAME 1.

Specifically, you can base your decision on whether the Receiver decided to punish Player 2.

In GAME 2, **you** will make **two different choices**, one for each of the possible **Player 3** actions. Your choice that matches the action the **Receiver** actually took in GAME 1 will count for your bonus.

NOTE: This study involves REAL PEOPLE, and your decisions will determine how much bonus you and the Receiver actually receive.

Start of Block: dec1hid

How many pence would you like to send to a **Receiver** who, as **Player 3** in GAME 1...

Chose TO punish **Player 2**?

	0	2	4	6	8	10
Pence to send ()						

Start of Block: dec2hid

How many pence would you like to send to a **Receiver** who, as **Player 3** in GAME 1...

Chose NOT to punish **Player 2**?

	0	2	4	6	8	10
Pence to send ()						

Start of Block: demographics

Thank you!

Lastly, please provide your demographic information.

What is your gender?

○ Male (1)

○ Female (2)

○ Other (3)

○ Prefer not to say (4)

---

*

What is your age in years?

____

Start of Block: debrief

Debrief

Player 3 / the Receiver really was a Prolific participant, and your decisions really do determine your bonus and the bonus of the other player. However, Player 1 and Player 2 in the first game are not real.

Please email nicole.engeler.19@ucl.ac.uk or message on Prolific if you would like further information.

Thank you for your participation!

Please advance to the next page to submit your responses.

1.4 Helping Impact Checking (2.1)

Start of Block: Information and consent

This study has been approved by the UCL Ethics Board. Project ID: ICN-PWB-NR-23-10-20A.

The study is being run by Nicole Engeler (nicole.engeler.19@ucl.ac.uk) under the supervision of Professor Nichola Raihani (n.raihani@ucl.ac.uk) in the Department of Experimental Psychology at UCL.

In this study, you will play one or two games. Your decisions during these games will affect your bonus payment and the bonus payment of other Prolific players.

The study is estimated to last around X minutes.

All data will be handled according to the General Data Protection Regulations and will be kept anonymous. Only researchers working with the Raihani Lab will have access to datafiles containing identifying information (e.g. Prolific ID). Completely de-identified datafiles (e.g. no Prolific ID) will be shared online in accordance with open-science practices. If you do not consent to de-identified data being shared, please do not take part in this study. If you do not wish to complete this study, please return your submission.

Local Data Protection Privacy Notice

The controller for this project will be University College London (UCL). The UCL Data Protection Officer provides oversight of UCL activities involving the processing of personal data, and can be contacted at data-protection@ucl.ac.uk

The lawful basis that will be used to process your personal data is: 'Public task'

Your personal data will be processed so long as it is required for the research project. We will anonymise or pseudonymise the personal data you provide, and will endeavour to minimise the processing of personal data wherever possible.

If you are concerned about how your personal data is being processed, or if you would like to contact us about your rights, please contact UCL in the first instance at data-protection@ucl.ac.uk.

If you do not wish to participate, or if you change your mind during the course of the study, please return your submission.

Ethics Officer: ethics@ucl.ac.uk

Please only consent to taking part in this study if you agree with ALL of the following statements:
(1) I have read the information above and understand I can email the researchers with any questions.
(2) I understand that my personal information will be used for the purposes explained to me. I understand that according to data protection legislation, 'public task' will be the lawful basis for processing.
(3) I understand that all personal information will remain confidential and that my data gathered in this study will be stored anonymously and securely. It will not be possible to identify me in any publications.
(4) I understand that my anonymised research data may be shared with, and used by, others for future research (no one will be able to identify you when these data are shared).
(5) I understand that I am free to withdraw from the study without penalty if I so wish, simply by returning my submission.

○ I **consent** to take part in this study. (1)

○ I do **NOT consent** to take part in this study. (0)

Skip To: End of Survey If Please only consent to taking part in this study if you agree with ALL of the following statement... = I do NOT consent to take part in this study.

Please enter your Prolific ID

____

Start of Block: Player A – Game 1

In addition to the base payment (£0.90) for participating in this study, you can earn a **bonus** based on your interaction with other Prolific participants during *two different games*.

Although some aspects of the two games are similar, in the first game you will be one of THREE players, and in the second game you will be one of TWO players.

GAME 1

GAME 1 is a three-player game. **You will be Player 3.**

Player 1 and **Player 2** have already read all the rules and made their decisions, and are now waiting for yours.

In GAME 1, each player starts with the following amounts:
Player 1: £0.10

Player 2: £0.00
You: £0.10

Player 1 has to choose how much, if anything, to send to **Player 2**.
Any amount sent to **Player 2** is tripled (e.g. for every 1p **Player 1** sends, **Player 2** receives 3p).
Player 2 then has to choose how much, if anything, to return to **Player 1**.

For instance, if **Player 1** sends £0.10 to Player 2, **Player 2** receives £0.30.
If **Player 2** returns half, both players earn £0.15 and both have more than **Player 1** started with.
If **Player 2** returns nothing, Player 2 keeps £0.30 and **Player 1** earns no bonus.

Players can also send or return any intermediate amount.

GAME 1

Player 1 chose to send all £0.10 to Player 2, and therefore Player 2 received £0.30.

Player 2 chose to keep everything (£0.30) and to return nothing (£0.00).

As a result, Player 1 will now receive no bonus (£0.00) and Player 2 will receive a bonus of £0.30.

You (Player 3) start this game with £0.10, and **can pay a cost to help Player 1 by increasing their bonus**. If you decide to help, the cost to yourself will be **£0.05**. (**Player 2**'s bonus will not be affected by your decision.)

If you choose to help **Player 1**, **you** will keep £0.05. If you choose NOT to help, **you** will keep all £0.10.

However, **you DO NOT know how much helping will add to Player 1's bonus**, only that it is somewhere between £0.01 and £0.30.

Example: If the impact of helping turned out to be £0.01, **Player 1** would finish with £0.01.
Example: If the impact of helping turned out to be £0.30, **Player 1** would finish with £0.30.

*While you currently do NOT know how much helping would impact **Player 1**, you can request to find out before deciding whether to help.*

So, **you** must decide:
1) Whether or not to find out how much helping would impact **Player 1**
2) Whether or not to help **Player 1**

Please answer the following questions, to make sure you understand GAME 1. **You MUST answer ALL questions correctly to receive your bonus!**

Imagine that you are deciding whether or not to help **Player 1**. Which *decision* that **you** make will result in the **highest** possible bonus for **you**?

- You deciding to help (0)
- You deciding NOT to help (1)
- Either decision could result in a higher bonus than the other decision, it depends on what the impact of helping is (0)

Imagine that you are deciding whether or not to help **Player 1**. Which *decision* that **you** make will result in the **highest** possible bonus for **Player 1**?

- You deciding to help (1)
- You deciding NOT to help (0)
- Which decision results in the highest bonus depends on what the impact of helping is (0)

Imagine that you are deciding whether or not to help **Player 1**. Which *decision* that **you** make will result in the **lowest** possible bonus for **Player 2**?

- You deciding to help (0)
- You deciding NOT to help (0)
- Your decision does not affect Player 2's bonus (1)

What are the two choices that you have?

- Whether to find out the impact of helping, and whether to help Player 1 (1)
- Whether to help Player 1 if the impact of helping is large, and whether to help Player 1 if the impact of helping is small (0)
- Whether to help Player 1, and how much to help Player 1 (0)

Start of Block: Player A – Game 2

GAME 2

After GAME 1, you will play GAME 2, which is a **two-player** game.

You will play GAME 2 with a NEW player who did NOT participate in GAME 1.

You will be the “**Receiver**". The new player will be the "**Sender**". There will be no further players.

In GAME 2:
- The **Sender** starts with £0.10
- The **Sender** chooses how much, if anything, to send to you (the **Receiver**)
- Any money sent to **you** is tripled: for every 1p the **Sender** sends, **you** receive 3p
- **You** then choose how much, if anything, to return to the **Sender**. You can return nothing, any intermediate amount, or everything.

If the **Sender** sends all £0.10, **you** will receive £0.30. If **you** return half of the £0.30, **you** and the **Sender** will both earn £0.15, and will both have more than the **Sender** started with.

But if **you** return £0.00, you will earn £0.30, while the **Sender** will earn £0.00.

So, the ***Sender*** *can gain or lose money by sending you money, depending on how much* ***you*** *return.*

Please answer the following questions, to make sure you understand GAME 2. **You MUST answer ALL questions correctly to receive your bonus!**

Imagine that the Sender is deciding how much to send to you. Which of the ***Sender's*** *decisions* will result in the **Sender** earning the highest payoff?

- The Sender deciding to send nothing (0)
- The Sender deciding to send everything (0)
- Which decision results in the highest payoff depends on how much you decide to return to the Sender (1)

Imagine that you are deciding how much to return to the Sender. Which of ***your*** *decisions* will result in **you** earning the highest payoff?

- You deciding to return nothing (1)
- You deciding to return everything (0)
- Which decision results in the highest payoff depends on how much the Sender sent you (0)

Did the **Sender** participate in GAME 1?

- Yes (0)
- No (1)

Start of Block: Player A Game 2 OBSERVABLE

Although the **Sender** did NOT participate in GAME 1, the **Sender** can base the decision of how much money to send to you on how **you** behaved in GAME 1.

Specifically, the Sender can base this decision on whether you decided to help Player 1, as well as whether you decided to find out the impact of helping.

Please answer the following question, to make sure you understand. You MUST answer ALL questions correctly to receive your bonus!

When deciding how much to send to **you**, what information can the Sender base this decision on?

- Nothing (0)
- Whether you helped Player 1 (0)
- Whether you decided to find out the impact of helping (0)
- Whether you helped Player 1, AND whether you decided to find out the impact of helping (1)

GAME 1

Helping entails **you** paying £0.05 to increase **Player 1**'s £0.00 bonus. The impact of helping is somewhere between £0.01 and £0.30, but you do not know exactly how much.

NOTE: This study involves REAL PEOPLE. *You really do have the chance to find out the impact of helping, and the Sender really does get to base their sending decision on whether you decide to find out*.

Would you like to find out how much helping impacts Player 1's bonus?

- Yes (1)
- No (0)

Start of Block: Checked Impact (A)

The impact of helping is £0.15.

Right now **you** have a £0.10 bonus. You know that helping would cost **you** £0.05 to increase **Player 1**'s £0.00 bonus by £0.15.

Would you like to pay the cost to help Player 1?

- ◯ Yes (1)
- ◯ No (0)

Start of Block: NO look (A)

You decided not to find out what the impact of helping is.

Right now **you** have a £0.10 bonus. You know that helping would cost **you** £0.05. Helping would add somewhere between £0.01 and £0.30 to **Player 1**'s £0.00 bonus.

Would you like to pay the cost to help Player 1?

- ◯ Yes (1)
- ◯ No (0)

Start of Block: Player A Game 2 HIDDEN

Although the **Sender** did NOT participate in GAME 1, the Sender can base the decision of how much money to send to you on how **you** behaved in GAME 1.

Specifically, the Sender can base this decision on whether you decided to help Player 1 (but *NOT* on whether you decided to find out the impact of helping).

When deciding how much to send to you, what information can the Sender base this decision on?

◯ Nothing (0)

◯ Whether you helped Player 1 (1)

◯ Whether you decided to find out the impact of helping (0)

◯ Whether you helped Player 1, AND whether you decided to find out the impact of helping (0)

X→

GAME 1

Helping entails **you** paying £0.05 to increase **Player 1's** £0.00 bonus. The impact of helping is somewhere between £0.01 and £0.30, but you do not know exactly how much.

NOTE: This study involves REAL PEOPLE. *You really do have the chance to find out the impact of helping.*

Would you like to find out how much helping impacts Player 1's bonus?

◯ Yes (1)

◯ No (0)

Start of Block: Player A Game 2 Decisions

GAME 2

The **Sender** will decide how much of their £0.10 to send to you.
You will decide how much to return to the Sender.

Specifically, you will decide the percentage of pence you would like to return.

For example, if **you** decide to return 40% of the pence you receive...
- If the **Sender** sends £0.00 (and you receive £0.00) **you** will return £0.00
- If the **Sender** sends £0.10 (and you receive £0.30) **you** will return £0.12

REMINDER: This study involves REAL PEOPLE, and your decision will determine how much bonus

you and the Sender actually receive.

What percentage would you like to return?

	0 10 20 30 40 50 60 70 80 90 100
Percentage to return ()	

Start of Block: Player B – Game 1

In addition to the base payment (£0.90) for participating in this study, you can earn a **bonus** based on your interaction with other Prolific participants during *two different games*.

You will ONLY take part in the second game, and will NOT play in the first game. However, we would like you to read about and understand both games.

GAME 1

GAME 1 is a three-player game.

Each player starts with the following amounts:
Player 1: £0.10
Player 2: £0.00
Player 3: £0.10

Player 1 has to choose how much, if anything, to send to **Player 2**.
Any amount sent to **Player 2** is tripled (e.g. for every 1p **Player 1** sends, **Player 2** receives 3p).
Player 2 then has to choose how much, if anything, to return to **Player 1**.

For instance, if **Player 1** sends £0.10 to Player 2, **Player 2** receives £0.30. If **Player 2** returns half, both players earn £0.15 and both have more than **Player 1** started with.
If **Player 2** returns nothing, then **Player 2** keeps £0.30 and **Player 1** earns no bonus.
Players can also send or return any intermediate amount.

Player 1 and Player 2 have already read all the rules and made their decisions.

GAME 1

Player 1 chose to send all £0.10 to Player 2, and therefore Player 2 received £0.30.

Player 2 chose to keep everything (£0.30) and to return nothing (£0.00).

As a result, Player 1 will now receive no bonus (£0.00) and Player 2 will receive a bonus of £0.30.

Player 3 *now has the chance to pay a cost to help* ***Player 1*** *by increasing their bonus.* (**Player 2**'s bonus will not be affected by Player 3's decision.)

If **Player 3** helps, the cost to themselves will be £0.05. If **Player 3** does NOT help, they will keep all £0.10.

However, **Player 3 DOES NOT know how much helping will add to Player 1's bonus**, only that it is somewhere between £0.01 and £0.30.

Example: If the impact of helping turned out to be £0.01, **Player 1** would finish with £0.01.
Example: If the impact of helping turned out to be £0.30, **Player 1** would finish with £0.30.

While ***Player 3*** *initially does NOT know how much help impacts* ***Player 1***, ***Player 3*** *can request to find out before deciding whether to help.*

So, **Player 3** must decide:
1) Whether or not to find out how much helping would impact **Player 1**
2) Whether or not to help **Player 1**

Please answer the following questions, to make sure you understand GAME 1. **You MUST answer ALL questions correctly to receive your bonus!**

X→

Imagine that Player 3 is deciding whether or not to help **Player 1**. Which of ***Player 3**'s decisions* will result in the **highest** possible bonus for **Player 3**?

○ Player 3 deciding to help (0)

○ Player 3 deciding NOT to help (1)

○ Either decision could result in a higher bonus than the other decision, it depends on what the impact of helping is (0)

Imagine that Player 3 is deciding whether or not to help **Player 1**. Which of **Player 3**'s decision will result in the **highest** possible bonus for **Player 1**?

- Player 3 deciding to help (1)
- Player 3 deciding NOT to help (0)
- Which decision results in the highest bonus depends on what the impact of helping is (0)

Imagine that Player 3 is deciding whether or not to help **Player 1**. Which of **Player 3**'s decision will result in the **lowest** possible bonus for **Player 2**?

- Player 3 deciding to help (0)
- Player 3 deciding NOT to help (0)
- Player 3's decision does not affect Player 2's bonus (1)

What are the two choices that **Player 3** has?

- Whether to find out the impact of helping, and whether to help Player 1 (1)
- Whether to help Player 1 if the impact of helping is large, and whether to help Player 1 if the impact of helping is small (0)
- Whether to help Player 1, and how much to help Player 1 (0)

Start of Block: Player B – Game 2

GAME 2

GAME 2 is a two-player game. *You will play this game with **Player 3** from GAME 1.*

You will be the "**Sender**". **Player 3** will be the "**Receiver**".

In GAME 2:

- You (the Sender) start with £0.10
- You then choose how much, if anything, to send to the Receiver
- Any money you send to the Receiver is tripled: for every 1p you send, the Receiver receives 3p
- The Receiver then chooses how much, if anything, to return to you. The Receiver can return nothing, any intermediate amount, or everything.

If you send all £0.10, the Receiver will receive £0.30. If the Receiver returns half of the £0.30, you and the Receiver will both earn £0.15, and will both have more than you started with.

But if the Receiver returns nothing, they will earn £0.30, while you will earn nothing.

So, you can gain or lose money by sending money to the Receiver, depending on how much the Receiver returns.

Please answer the following questions, to make sure you understand GAME 2. **You MUST answer ALL questions correctly to receive your bonus!**

X→

Imagine that you are deciding how much to send to the Receiver. Which of *your decisions* will result in the **highest** possible bonus for you?

◯ You deciding to send nothing (0)

◯ You deciding to send everything (0)

◯ Which decision results in the highest bonus depends on how much the Receiver decides to return to you (1)

X→

Imagine that the Receiver is deciding how much to return to you. Which of the Receiver's decisions will result in the **highest** possible bonus for the Receiver?

◯ The Receiver deciding to return nothing (1)

◯ The Receiver deciding to return everything (0)

◯ Which decision results in the highest bonus depends on how much you sent to the Receiver (0)

Did the **Receiver** participate in GAME 1?

- ◯ Yes (1)
- ◯ No (0)

Start of Block: B G2 OBSERV

GAME 2

You currently have a £0.10 bonus.

You will now decide how much, if anything, to send to the Receiver.

You can base this decision on how the Receiver behaved as Player 3 in GAME 1. Specifically, you can base your decision both on whether the Receiver decided to find out what the impact of helping was, and whether the Receiver decided to help Player 1.

In GAME 1, the impact of helping turned out to be £0.15. So, helping entailed Player 3 sacrificing £0.05 to give £0.15 to Player 1. But Player 3 only knew what the impact of helping was if they decided to find out.

In GAME 2, you will make ***four different choices***, one for each of the possible Player 3 decisions. Your choice that matches with the decision the Receiver actually made in GAME 1 will count for your bonus.

NOTE: This study involves REAL PEOPLE, and your decisions will determine how much bonus you and the Receiver actually receive.

Start of Block: Bdec1OBS

How many pence would you like to send to a Receiver who, as Player 3 in GAME 1...

Chose TO help, WITHOUT finding out how much helping would impact Player 1?

	0	2	4	6	8	10
Pence to send ()						

Start of Block: Bdec2OBS

How many pence would you like to send to a **Receiver** who, as **Player 3** in GAME 1…

Chose NOT to help, WITHOUT finding out how much helping would impact **Player 1**?

	0	2	4	6	8	10
Pence to send ()						

Start of Block: Bdec3OBS

How many pence would you like to send to a **Receiver** who, as **Player 3** in GAME 1…

Chose TO help, WITH finding out how much helping would impact **Player 1**?

	0	2	4	6	8	10
Pence to send ()						

Start of Block: Bdec4OBS

How many pence would you like to send to a **Receiver** who, as **Player 3** in GAME 1…

Chose NOT to help, WITH finding out how much helping would impact **Player 1**?

	0	2	4	6	8	10
Pence to send ()						

Start of Block: B G2 HIDDEN

GAME 2

You currently have a £0.10 bonus.

You will now decide how much, if anything, to send to the **Receiver**.

You can base this decision on how the Receiver behaved as Player 3 in GAME 1. Specifically, you can base your decision on whether the Receiver decided to help Player 1.

In GAME 1, the impact of helping turned out to be £0.15. So, helping entailed **Player 3** sacrificing

£0.05 to give £0.15 to **Player 1**. But **Player 3** only knew what the impact of helping was if they decided to find out.

In GAME 2, **you** will make **two different choices**, one for each of the possible **Player 3** decisions. **Your** choice that matches with the decision the **Receiver** actually made in GAME 1 will count for your bonus.

NOTE: This study involves REAL PEOPLE, and your decisions will determine how much bonus you and the Receiver actually receive.

Start of Block: Bdec1hid

How many pence would you like to send to a **Receiver** who, as **Player 3** in GAME 1...

Chose TO help **Player 1**?

	0	2	4	6	8	10
Pence to send ()						

Start of Block: Bdec2hid

How many pence would you like to send to a **Receiver** who, as **Player 3** in GAME 1...

Chose NOT to help **Player 1**?

	0	2	4	6	8	10
Pence to send ()						

Start of Block: demographics

Thank you!

Lastly, please provide your demographic information.

What is your gender?

- ○ Male (1)
- ○ Female (2)
- ○ Other (3)
- ○ Prefer not to say (4)

---

*

What is your age in years?

____

Start of Block: debrief

Debrief

Player 3 / the Receiver and the Sender really are Prolific participants, and your decisions really do determine your bonus and the bonus of the other player. However, Player 1 and Player 2 in the first game are not real.

Please email nicole.engeler.19@ucl.ac.uk or message on Prolific if you would like further information.

Thank you for your participation!

Please advance to the next page to submit your responses.

SUPPLEMENTARY NOTES 1

	All Participants			Excellent Comprehension		
	**Original Scale**	**Scale x 0.5**	**Scale x 1.5**	**Original Scale**	**Scale x 0.5**	**Scale x 1.5**
H1.1	**71.28**	54.86	62.34	**309.86**	132.18	350.35
H1.2a	**6972.31**	3878.78	6754.62	**1501.72**	588.75	1730.66
H1.2b	**2.16**	2.16	2.16	**5.42**	5.42	5.42
H2.1	**6.00**	2.31	8.61	**8.97**	2.38	22.42
H2.2a	**4.78**	1.89	5.49	**0.72**	1.00	0.81
H2.2b	**0.39**	0.74	0.26	**0.17**	0.34	0.12
H3	**0.10**	0.30	0.19	**0.31**	0.54	0.21
H4	**140.10**	659.85	41.74	**20.22**	17.30	17.07
H5.1	**3.86**	4.11	3.07	**0.67**	0.91	0.51
H5.2	**0.60**	0.93	0.43	**5.89**	4.73	5.28
H6.1	**1.53**	1.25	1.46	**1.87**	1.28	2.39
H6.2	**4.31**	1.88	6.52	**4.90**	1.70	10.11
H7.1	**0.68**	0.80	0.42	**0.59**	0.81	0.46
H7.2a	**0.80**	0.87	0.56	**1.20**	1.13	1.13
H7.2b	**0.26**	0.45	0.19	**0.34**	0.58	0.23
H8.1	**1.16**	1.61	0.86	**2.10**	2.22	1.73
H8.2a	**1.17**	1.57	0.84	**0.66**	0.90	0.50
H8.2b	**0.28**	0.53	0.17	**0.24**	0.43	0.16
H9.1	**18.29**	18.29	18.29	**2.38**	2.38	2.38
H9.2a	**0.14**	0.14	0.14	**0.17**	0.17	0.17
H9.2b	**0.37**	0.62	0.26	**0.31**	0.54	0.22
H10	**0.75**	0.02	0.14	**0.39**	0.62	0.26
H11.1	**5.05**	3.25	5.00	**10.87**	4.04	15.00
H11.2	**0.48**	0.75	0.35	**0.86**	1.00	0.73
H12.1	**2.13**	2.53	1.43	**5.89**	4.83	5.07
H12.2	**0.79**	1.01	0.49	**0.57**	0.80	0.42
H13.1	**8.59**	8.59	8.59	**0.95**	0.95	0.95
H13.2	**0.13**	0.13	0.13	**0.25**	0.25	0.25
H14.1	**97.33**	97.33	97.33	**7746.67**	7746.67	7746.67
H14.2a	**0.17**	0.17	0.17	**0.31**	0.31	0.31
H14.2b	**0.14**	0.14	0.14	**0.49**	0.49	0.49
H15.1	**0.56**	0.56	0.56	**0.27**	0.27	0.27
H15.2a	**1.98**	1.98	1.98	**1.03**	1.03	1.03
H15.2b	**0.01**	0.01	0.01	**0.03**	0.03	0.03
H16	**0.24**	0.24	0.24	**0.16**	0.16	0.16
H17	**0.33**	0.33	0.33	**0.14**	0.14	0.14
H18	**0.17**	0.17	0.17	**0.48**	0.48	0.48
H19.1	**0.44**	0.44	0.44	**0.62**	0.62	0.62
H19.2	**0.12**	0.12	0.12	**0.24**	0.24	0.24
H20.1	**1.02**	1.02	1.02	**0.44**	0.44	0.44
H20.2	**0.08**	0.08	0.08	**0.12**	0.12	0.12
Add. 1	**0.98**	0.82	0.30	**0.65**	0.84	0.42
Add. 2	**3.45**	3.45	3.45	**0.46**	0.46	0.46

Supplementary Table 1: Bayes Factor Sensitivity Analysis. This table presents Bayes factors for all hypotheses, both when all participants and only those with excellent comprehension are included. Bayes factors produced from the original scale are bolded, followed by results from scale adjustments (prior scale of 0.5 times and 1.5 times the original value).
