# Supplementary material for: Acting without considering personal costs signals trustworthiness in helpers but not punishers
Source: Commun Psychol. 2024 May 24;2:47. doi: 10.1038/s44271-024-00092-7 (PMC11332106; doi:10.1038/s44271-024-00092-7)
Supplement: Supplementary file 3 — Reporting Summary [file 44271_2024_92_MOESM3_ESM.pdf]

nature portfolio

Corresponding author(s): Nicole C Engeler

Last updated by author(s): Mar 21, 2024

Reporting Summary

Nature Portfolio wishes to improve the reproducibility of the work that we publish. This form provides structure for consistency and transparency in reporting. For further information on Nature Portfolio policies, see our Editorial Policies and the Editorial Policy Checklist.

Statistics

For all statistical analyses, confirm that the following items are present in the figure legend, table legend, main text, or Methods section.

n/a	Confirmed	
☐	☒	The exact sample size (*n*) for each experimental group/condition, given as a discrete number and unit of measurement
☐	☒	A statement on whether measurements were taken from distinct samples or whether the same sample was measured repeatedly
☐	☒	The statistical test(s) used AND whether they are one- or two-sided<br>*Only common tests should be described solely by name; describe more complex techniques in the Methods section.*
☐	☒	A description of all covariates tested
☐	☒	A description of any assumptions or corrections, such as tests of normality and adjustment for multiple comparisons
☐	☒	A full description of the statistical parameters including central tendency (e.g. means) or other basic estimates (e.g. regression coefficient) AND variation (e.g. standard deviation) or associated estimates of uncertainty (e.g. confidence intervals)
☐	☒	For null hypothesis testing, the test statistic (e.g. *F*, *t*, *r*) with confidence intervals, effect sizes, degrees of freedom and *P* value noted<br>*Give P values as exact values whenever suitable.*
☐	☒	For Bayesian analysis, information on the choice of priors and Markov chain Monte Carlo settings
☐	☒	For hierarchical and complex designs, identification of the appropriate level for tests and full reporting of outcomes
☐	☒	Estimates of effect sizes (e.g. Cohen's *d*, Pearson's *r*), indicating how they were calculated

Our web collection on statistics for biologists contains articles on many of the points above.

Software and code

Policy information about availability of computer code

Data collection	No software was used to collect the data.
Data analysis	Analysis was conducted in R, notably using the lmtest, BayesFactor and brms packages.

For manuscripts utilizing custom algorithms or software that are central to the research but not yet described in published literature, software must be made available to editors and reviewers. We strongly encourage code deposition in a community repository (e.g. GitHub). See the Nature Portfolio guidelines for submitting code & software for further information.

Data

Policy information about availability of data

All manuscripts must include a data availability statement. This statement should provide the following information, where applicable:

- Accession codes, unique identifiers, or web links for publicly available datasets
- A description of any restrictions on data availability
- For clinical datasets or third party data, please ensure that the statement adheres to our policy

All study data and materials, as well as the laboratory log are available on OSF under this link: https://osf.io/y2hgu/.

Human research participants

Policy information about studies involving human research participants and Sex and Gender in Research.

Reporting on sex and gender	Participants self-reported their gender. This information is not relevant to our study design, therefore gender is only reported as demographic information. Experiment 1: 1381 women and 1207 men (17 identified as 'other' and 7 preferred not to say); Experiment 2: 1519 women and 1071 men (18 identified as 'other' and 7 preferred not to say), Experiment 3: 1598 women and 990 men (22 identified as 'other', and 2 preferred not to say); Experiment 4 1645 women and 949 men (21 'other', 2 preferred not to say); Experiment 5: 1581 women and 1002 men (20 'other', and 9 preferred not to say). Participants provided informed consent and were compensated at a rate of £9 per hour. No data on race / ethnicity was collected.
Population characteristics	Participants were aged 18 and above, from the UK (so that the currency specifications of the economic games they played were familiar), and fluent in English. No demographic information was a covariate-relevant characteristic.
Recruitment	Participants were recruited via Prolific. Participants were invited to take part if they had previously indicated on Prolific that they (i) are aged 18 or above, (ii) are from the UK, (iii) are fluent in English, (iv) have the maximum approval rate of 100 (reduced in one-unit increments to 97 when more participants were needed), and (v) selected "Yes, I would be comfortable to take part in such a study" to the question "Would you be happy to take part in a study where you are intentionally given inaccurate information about other participants and the study? You would be debriefed after the study". To avoid participants taking part in more than one experiment, we launched the experiments in sequence, and allowed only new participants to take part.
Ethics oversight	UCL Ethics Board

Note that full information on the approval of the study protocol must also be provided in the manuscript.

Field-specific reporting

Please select the one below that is the best fit for your research. If you are not sure, read the appropriate sections before making your selection.

☐ Life sciences ☒ Behavioural & social sciences ☐ Ecological, evolutionary & environmental sciences

For a reference copy of the document with all sections, see nature.com/documents/nr-reporting-summary-flat.pdf

Behavioural & social sciences study design

All studies must disclose on these points even when the disclosure is negative.

Study description	This is a quantitative expermental study.
Research sample	Participants were Prolific users, aged 18 and above, from the UK (so that the currency specifications of the economic games were familiar), and fluent in English. The sample is not representative.
Sampling strategy	Our power calculation was conducted in R using the package 'pwr' with the 'pwr.f2.test' function. We used a power of 0.95 with a 0.05 significance level and a one numerator degree of freedom (u, the number of coefficients in the model without the intercept). While estimating the required sample size, we referred to Jordan et al.'s supplementary materials for effect sizes, but specific effect sizes were not explicitly mentioned. We acknowledge that the available coefficients in their supplementary materials vary considerably, but generally produced small to medium effect sizes. As their study closely matches our experimental design, procedure, and research questions, we used an effect size of f2 = 0.02 in our power analysis. According to Cohen's guidelines, f2≥ 0.02 represents a small effect size. Because our main interests focus on third-party punishment rather than helping, we expect to find similar or smaller effect sizes. Nevertheless, we must acknowledge that our choice of f2 = 0.02 might be considered a heuristic approximation rather than a precise estimation based on a formal inspection of Jordan et al.'s results. Based on the model with the highest number of predictors (as n = v + p, with p being the number of predictors including the intercept and v the degrees of freedom for the denominator), a sample size of 653 would be needed. However, as each of the models upon which this calculation is based involves either a Player A or a Player B participant, taking part in one of five experiments, in either the process hidden or in the process observable condition, a sample size of 13,060 (i.e., 1,306 Player A - Player B pairs per experiment) is needed. As we could not predict how many participants would decide to punish/ help or not punish/ help, it was not possible to ascertain before data collection whether 95% power would be achieved for all analyses. Results that do not meet the power requirements are therefore interpreted as suggestive, pending confirmation in future research. Prolific predominantly uses convenience sampling (most study places are filled on a on a first-come, first-serve basis). However, Prolific has several mechanisms to fairly distribute study places among active participants.
Data collection	Data was collected online, using Qualtrics surveys.
Timing	All data collection was completed between 13th November 2023 and 24th November 2023 (total: 13073 participants).
Data exclusions	We used the "force response" feature in Qualtrics to ensure that we did not receive incomplete responses. Any exclusions were pre-

Data exclusions	registered. Responses by participants who failed more than one attention check were still included in the analyses. However, we re-ran the same analyses excluding those who failed more than one attention check, and used this version when it lead to significant differences in results. There were no duplicate responses to be removed.
Non-participation	Around 7% of participants who accepted the study on Prolific dropped out of the study, as indicated by how many participants returned the study on Prolific.
Randomization	Separate studies were conducted for helping and punishing behaviours. For the decision time study, participants had to be separately recruited for Player A vs Player B positions. For the other 4 studies, participants were randomly allocated Player A or Player B status. Decision process observability conditions were also be randomly allocated.

Reporting for specific materials, systems and methods

We require information from authors about some types of materials, experimental systems and methods used in many studies. Here, indicate whether each material, system or method listed is relevant to your study. If you are not sure if a list item applies to your research, read the appropriate section before selecting a response.

Materials & experimental systems

n/a	Involved in the study
☒	☐ Antibodies
☒	☐ Eukaryotic cell lines
☒	☐ Palaeontology and archaeology
☒	☐ Animals and other organisms
☒	☐ Clinical data
☒	☐ Dual use research of concern

Methods

n/a	Involved in the study
☒	☐ ChIP-seq
☒	☐ Flow cytometry
☒	☐ MRI-based neuroimaging
